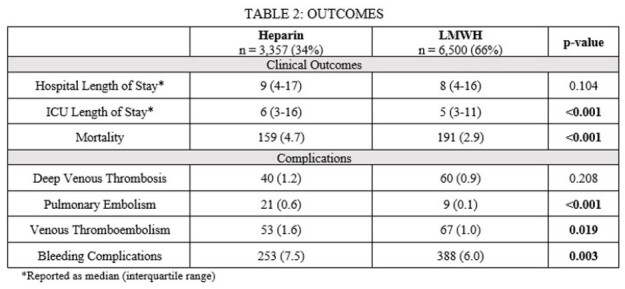# 707 Comparing VTE Prophylaxis Strategies in Thermal Injuries: Don't Get Burned by Heparin!

**DOI:** 10.1093/jbcr/irad045.183

**Published:** 2023-05-15

**Authors:** Christopher O'Neil, Walter Ramsey, Matthew Meece, Kenneth Proctor, Shevonne Satahoo, Joyce Kaufman, Louis Pizano, Carl Schulman

**Affiliations:** University of Miami, DeWitt Daughtry Family Department of Surgery , Miami, Florida; DeWitt Daughtry Family Department of Surgery, University of Miami, Miami, Florida; DeWitt Daughtry Family Department of Surgery, University of Miami, Miami, Florida; DeWitt Daughtry Family Department of Surgery, University of Miami, Miami, Florida; University of Miami, Miami, Florida; University of Miami, Miami, Florida; University of Miami, Miami, Florida; University of Miami-Jackson Memorial Hospital, Miami, Florida; University of Miami, DeWitt Daughtry Family Department of Surgery , Miami, Florida; DeWitt Daughtry Family Department of Surgery, University of Miami, Miami, Florida; DeWitt Daughtry Family Department of Surgery, University of Miami, Miami, Florida; DeWitt Daughtry Family Department of Surgery, University of Miami, Miami, Florida; University of Miami, Miami, Florida; University of Miami, Miami, Florida; University of Miami, Miami, Florida; University of Miami-Jackson Memorial Hospital, Miami, Florida; University of Miami, DeWitt Daughtry Family Department of Surgery , Miami, Florida; DeWitt Daughtry Family Department of Surgery, University of Miami, Miami, Florida; DeWitt Daughtry Family Department of Surgery, University of Miami, Miami, Florida; DeWitt Daughtry Family Department of Surgery, University of Miami, Miami, Florida; University of Miami, Miami, Florida; University of Miami, Miami, Florida; University of Miami, Miami, Florida; University of Miami-Jackson Memorial Hospital, Miami, Florida; University of Miami, DeWitt Daughtry Family Department of Surgery , Miami, Florida; DeWitt Daughtry Family Department of Surgery, University of Miami, Miami, Florida; DeWitt Daughtry Family Department of Surgery, University of Miami, Miami, Florida; DeWitt Daughtry Family Department of Surgery, University of Miami, Miami, Florida; University of Miami, Miami, Florida; University of Miami, Miami, Florida; University of Miami, Miami, Florida; University of Miami-Jackson Memorial Hospital, Miami, Florida; University of Miami, DeWitt Daughtry Family Department of Surgery , Miami, Florida; DeWitt Daughtry Family Department of Surgery, University of Miami, Miami, Florida; DeWitt Daughtry Family Department of Surgery, University of Miami, Miami, Florida; DeWitt Daughtry Family Department of Surgery, University of Miami, Miami, Florida; University of Miami, Miami, Florida; University of Miami, Miami, Florida; University of Miami, Miami, Florida; University of Miami-Jackson Memorial Hospital, Miami, Florida; University of Miami, DeWitt Daughtry Family Department of Surgery , Miami, Florida; DeWitt Daughtry Family Department of Surgery, University of Miami, Miami, Florida; DeWitt Daughtry Family Department of Surgery, University of Miami, Miami, Florida; DeWitt Daughtry Family Department of Surgery, University of Miami, Miami, Florida; University of Miami, Miami, Florida; University of Miami, Miami, Florida; University of Miami, Miami, Florida; University of Miami-Jackson Memorial Hospital, Miami, Florida; University of Miami, DeWitt Daughtry Family Department of Surgery , Miami, Florida; DeWitt Daughtry Family Department of Surgery, University of Miami, Miami, Florida; DeWitt Daughtry Family Department of Surgery, University of Miami, Miami, Florida; DeWitt Daughtry Family Department of Surgery, University of Miami, Miami, Florida; University of Miami, Miami, Florida; University of Miami, Miami, Florida; University of Miami, Miami, Florida; University of Miami-Jackson Memorial Hospital, Miami, Florida; University of Miami, DeWitt Daughtry Family Department of Surgery , Miami, Florida; DeWitt Daughtry Family Department of Surgery, University of Miami, Miami, Florida; DeWitt Daughtry Family Department of Surgery, University of Miami, Miami, Florida; DeWitt Daughtry Family Department of Surgery, University of Miami, Miami, Florida; University of Miami, Miami, Florida; University of Miami, Miami, Florida; University of Miami, Miami, Florida; University of Miami-Jackson Memorial Hospital, Miami, Florida

## Abstract

**Introduction:**

Burn patients are at high risk of venous thromboembolism (VTE) as well as bleeding complications, thus choosing the best option for thromboprophylaxis can be challenging. Previous studies have analyzed the preferred antithrombotic agent in trauma patients, but this has not been established in burns. We hypothesize that low molecular weight heparin (LMWH) is associated with better outcomes than unfractionated heparin.

**Methods:**

The Trauma Quality Improvement Project (TQIP) dataset was used to identify all patients with second- or third-degree burns that were administered VTE prophylaxis. Cases with missing data, < 24-hour hospital length of stay, other serious traumatic injuries (any AIS >3), interhospital transfer, history of kidney disease, or pre-hospital history of anticoagulant use were excluded. Bleeding complications were defined as hemorrhage control surgery, embolization procedures, or blood transfusion occurring after the initiation of thromboprophylaxis, consistent with other studies analyzing bleeding complications in TQIP. Rates of VTE and bleeding complications were compared between those who received heparin versus LMWH. Propensity score matching was performed to control for age, total body surface area percent (TBSA%) burned, and ICU admission (Table 1).

**Results:**

The analysis included 9,857 patients (69% male). Median age was 50 (34-62) years. LWMH was associated with fewer complications including pulmonary embolism, any VTE, and bleeding (Table 2). Initiation of thromboprophylaxis within 24 hours of arrival was associated with a lower risk of VTE (1.0% vs 2.1%, p< 0.001) without showing a significant difference in bleeding complications (6.4% vs 7.2%, p=0.206).

**Conclusions:**

This propensity score matched analysis of nearly ten thousand burn patients has demonstrated that low molecular weight heparin is associated with reduced risk of VTE and bleeding compared to heparin. Initiation of thromboprophylaxis within 24 hours is linked to lower rates of VTE. In burn patients without contraindications, thromboprophylaxis with LMWH should be started within 24 hours.

**Applicability of Research to Practice:**

Venous thromboembolism is an important complication in burn patients, which may negatively impact patient outcomes. These patients regularly receive DVT prophylaxis and it is important for their care team to be able to select the proper agents.